# Partner-delivered HIV self-test kits with and without financial incentives in antenatal care and index patients with HIV in Malawi: a three-arm, cluster-randomised controlled trial

**DOI:** 10.1016/S2214-109X(21)00175-3

**Published:** 2021-06-15

**Authors:** Augustine T Choko, Katherine Fielding, Cheryl C Johnson, Moses K Kumwenda, Richard Chilongosi, Rachel C Baggaley, Rose Nyirenda, Linda A Sande, Nicola Desmond, Karin Hatzold, Melissa Neuman, Elizabeth L Corbett

**Affiliations:** a TB–HIV Group, Malawi-Liverpool-Wellcome Clinical Research Programme, Chichiri, Blantyre, Malawi; b Department of Infectious Disease Epidemiology and MRC Tropical Epidemiology Group, London School of Hygiene & Tropical Medicine, London, UK; c Department of Global Health and Development, London School of Hygiene & Tropical Medicine, London, UK; d Department of Clinical Research, London School of Hygiene & Tropical Medicine, London, UK; e Global HIV, Hepatitis and STI Programme, WHO, Geneva, Switzerland; f Population Services International, Lilongwe, Malawi; g Department of HIV–AIDS, Ministry of Health, Lilongwe, Malawi; h Department of Clinical Sciences, Liverpool School of Tropical Medicine, Liverpool, UK; i Population Services International, Johannesburg, South Africa

## Abstract

**Background:**

Secondary distribution of HIV self-testing (HIVST) kits by patients attending clinic services to their partners could improve the rate of HIV diagnosis. We aimed to investigate whether secondary administration of HIVST kits, with or without an additional financial incentive, via women receiving antenatal care (ANC) or via people newly diagnosed with HIV (ie, index patients) could improve the proportion of male partners tested or the number of people newly diagnosed with HIV.

**Methods:**

We did a three-arm, open-label, pragmatic, cluster-randomised trial of 27 health centres (clusters), eligible if they were a government primary health centre providing ANC, HIV testing, and ART services, across four districts of Malawi. We recruited women (aged ≥18 years) attending their first ANC visit and whose male partner was available, not already taking ART, and not already tested for HIV during this pregnancy (ANC cohort), and people (aged ≥18 years) with newly diagnosed HIV during routine clinic HIV testing who had at least one sexual contact not already known to be HIV-positive (index cohort). Centres were randomly assigned (1:1:1), using a public selection of computer-generated random allocations, to enhanced standard of care (including an invitation for partners to attend HIV testing services), HIVST only, or HIVST plus a US$10 financial incentive for retesting. The primary outcome for the ANC cohort was the proportion of male partners reportedly tested, as ascertained by interview with women in this cohort at day 28. The primary outcome for the index cohort was the geometric mean number of new HIV-positive people identified per facility within 28 days of enrolment, as measured by observed HIV test results. Cluster-level summaries compared intervention with standard of care by intention to treat. This trial is registered with ClinicalTrials.gov, NCT03705611.

**Findings:**

Between Sept 8, 2018, and May 2, 2019, nine clusters were assigned to each trial arm, resulting in 4544 eligible women in the ANC cohort (1447 [31·8%] in the standard care group, 1465 [32·2%] in the HIVST only group, and 1632 [35·9%] in HIVST plus financial incentive group) and 708 eligible patients in the index cohort (234 [33·1%] in the standard care group, 169 [23·9%] in the HIVST only group, and 305 [42·9%] in the HIVST plus financial incentive group). 4461 (98·2%) of 4544 eligible women in the ANC cohort and 645 (91·1%) of 708 eligible patients in the index cohort were recruited, of whom 3378 (75·7%) in the ANC cohort and 439 (68·1%) in the index cohort were interviewed after 28 days. In the ANC cohort, the mean proportion of reported partner testing per cluster was 35·0% (SD 10·0) in the standard care group, 73·0% in HIVST only group (13·1, adjusted risk ratio [RR] 1·71, 95% CI 1·48–1·98; p<0·0001), and 65·2% in the HIVST plus financial incentive group (11·6, adjusted RR 1·62, 1·45–1·81; p<0·0001). In the index cohort, the geometric mean number of new HIV-positive sexual partners per cluster was 1·35 (SD 1·62) for the standard care group, 1·91 (1·78) for the HIVST only group (incidence rate ratio adjusted for number eligible as an offset in the negative binomial model 1·65, 95% CI 0·49–5·55; p=0·3370), and 3·20 (3·81) for the HIVST plus financial incentive group (3·11, 0·99–9·77; p=0·0440). Four self-resolving, temporary marital separations occurred due to disagreement in couples regarding HIV self-test kits.

**Interpretation:**

Although administration of HIVST kits in the ANC cohort, even when offered alongside a financial incentive, did not identify significantly more male patients with HIV than did standard care, out-of-clinic options for HIV testing appear more acceptable to many male partners of women with HIV, increasing test uptake. Viewed in the current context, this approach might allow continuation of services despite COVID-19-related lockdowns.

**Funding:**

Unitaid, through the Self-Testing Africa Initiative.

**Research in context**

**Evidence before this study**

We searched PubMed on March 15, 2021, with no language restrictions, for titles and abstracts published between Jan 1, 1980, and March 15, 2021, using the search terms: (“HIV self-testing”[Title/Abstract]) OR (“HIV self testing”[Title/Abstract]), and found 483 records. Women are far more likely to attend HIV clinics for testing than are men. Furthermore, pregnancy is a period of high risk for HIV acquisition and transmission for fetuses and for sexual partners. Partner testing during pregnancy is recommended by WHO and is part of national guidelines, but implementation and uptake remains minimal. Studies in Malawi and Kenya have found increased uptake of testing among male partners of pregnant women when kits are offered in antenatal care; however, these studies have been done outside of real-world settings and have collected data on few outcomes. Previous studies on HIV self-testing (HIVST) among index partners have been limited to partners of patients receiving antiretroviral therapy who were recruited from outpatient facilities in Malawi. One such study found increased uptake of testing but poor linkage to care following HIVST implementation and, despite generally good acceptability of these tests, men aged 30 years and older reported challenges doing the test.

**Added value of this study**

Unlike previous studies that were implemented by research staff, in this study we show that it is feasible to integrate distribution of HIVST kits and management of clients with government personnel. Secondary distribution of HIVST kits by pregnant women or index patients to their male partners or sexual contacts improved uptake of testing in a pragmatic setting, which adds novelty relative to previous work. Although we found no significant difference in the numbers of new HIV diagnoses, our study extends knowledge on novel strategies for contact tracing and testing for newly diagnosed clinic attendees who are HIV-positive (so-called index testing).

**Implications of all the available evidence**

Our results show that secondary distribution of HIVST kits is an effective strategy that can support targeting of hard-to-reach groups, such as men and sexual contacts of HIV-positive patients. We show that secondary distribution of HIVST kits can be readily integrated into routine clinic activities with few adverse events; therefore, they should now be considered regional priority. The results suggest an urgent need to optimise materials to enable users to get correct results under secondary distribution, especially in poor rural communities. In any case, novel strategies for maintaining HIV services are needed during the COVID-19 pandemic, and approaches based on HIVST have the major advantage of not requiring direct interaction between contacts and providers until confirmatory testing and enrolment into HIV care. HIVST can meet unique programmatic needs and global HIV testing targets, both during the COVID-19 pandemic and in the long term, and should be implemented routinely in all settings with high HIV prevalence.

## Introduction

Testing plays a key role in control of infectious diseases, including HIV, and it is crucial for diagnosis, treatment, and prevention. Men aged 30 years and older, adolescents, the most economically disadvantaged people, and key populations (eg, men who have sex with men, sex workers, etc) have a high risk of undiagnosed HIV, and they report facing substantial barriers to standard facility-based HIV testing services.^1^ HIV self-testing (HIVST) provides a convenient, intrinsically confidential, and often preferred testing approach that can bypass facility access barriers, such as high indirect and opportunity costs, worries about confidentiality or reliability of routine services, and anticipated stigma.^2^ HIVST was fully endorsed by WHO as a recommended approach to providing HIV testing services in November, 2019, and it is promoted as an important tool for reaching HIV elimination targets in all global regions by international disease control initiatives (eg, the US Agency for International Development–President's Emergency Plan for AIDS Relief).^1^, ^3^ HIVST also provides an evidence-based option to maintain HIV testing services, despite disruption to routine service delivery from the COVID-19 pandemic.^1^

Among the most promising HIVST delivery strategies is secondary distribution, whereby patients attending clinic services take kits home for their sexual partners.^4^, ^5^, ^6^ Secondary distribution from antenatal care (ANC) clinics is safe,^7^ acceptable to both partners, and the only approach tried, to date, that results in high uptake of HIV testing by male partners of pregnant women,^4^, ^5^, ^6^ which is a key component of strategies for eliminating paediatric HIV.^8^ Although less well defined, secondary distribution of HIVST kits also holds promise as an additional way to test the sexual partners of people with newly diagnosed HIV (so-called index testing).^2^ Index testing provides a high yield of newly diagnosed HIV-positive people globally;^9^ however, this approach was only endorsed in 2016–19 by HIV programmes because of concerns around confidentiality, stigma, and the safety of index patients and their partners, as well as feasibility due to existing laws and policies, and limited resources to support providers with ongoing training, monitoring, and supervision.

Malawi is a country in southeast Africa that has a high HIV prevalence, with an estimated adult prevalence of 9·2%.^10^ Undiagnosed HIV and the incidence of new HIV infection in adults and neonates have been substantially reduced by a highly efficient and pragmatic HIV testing and antiretroviral therapy (ART) programme, with an estimated 90·0% of people living with HIV diagnosed and 87·0% of those receiving ART in 2018.^10^ Nevertheless, providing HIV testing to male partners of pregnant women during pregnancy has remained an important gap, contributing to new HIV infections in pregnant and breastfeeding women that now account for a high proportion of all perinatal HIV infections in Malawi.^11^ Index testing has been routinely implemented by use of patient referral or an invitation by letter (eg, family referral slip) for all partners and family members of people with newly diagnosed HIV to attend for HIV testing, without further active follow-up.^12^

Financial incentives address economic barriers associated with HIV testing and care, including direct and opportunity costs, especially for men.^13^ A growing body of literature suggests that the use of financial incentives increases HIV testing and clinic attendance for HIV services, including ART initiation, especially in hard-to-reach groups such as men.^4^, ^14^ However, in resource-limited settings with heightened amounts of corruption, few delivery platforms, and personal risk to staff, use of financial incentives is largely unsupported by policy makers.

In this study, set in Malawi, we aimed to investigate whether secondary administration of HIVST kits, in the presence or absence of an additional financial incentive, via women receiving ANC could improve the proportion of male partners being tested. We also aimed to investigate whether secondary administration of these test kits, with or without a financial incentive, via people who had been newly diagnosed with HIV (ie, index patients) could improve the number of people newly diagnosed with HIV.

## Methods

### Study design and participants

We did a three-arm, open-label, pragmatic, cluster-randomised trial of 27 government health centres (clusters) across four districts (Blantyre, Zomba, Thyolo, and Mulanje) of Malawi. Centres were eligible to be a cluster if they were a government primary health clinic or centre providing ANC, HIV testing, and ART services. The minimum distance between any two clusters was 5 km, which substantially reduced or eliminated any potential contamination. Each cluster enrolled women attending ANC (ANC cohort) and people with newly diagnosed HIV during routine clinic HIV testing (index cohort) into one of three trial arms (standard of care, HIVST only, and HIVST plus financial incentive). Each health centre recruited participants for both cohorts. HIVST kits were provided by routine health staff after verbal consent was obtained, given that all components were already national policy. Leaflets for recipients explained that data would be used for a research study. Written informed consent was obtained from all participants within clusters assigned to the HIVST plus financial incentive group, as well as their sexual partners, who all took part in a diagnostic accuracy substudy. Ethics approval was obtained from the Malawi College of Medicine Research Ethics Committee (P.02/18/2352) and London School of Hygiene & Tropical Medicine Ethics Committee (14916). Although there is potential for harm resulting from false positive or false negative HIVST results and coercion, the evidence indicates negligible social harms from introducing HIVST.^7^, ^15^

Eligible women attending ANC were enrolled into the ANC clinic cohort unless newly diagnosed with HIV, in which case they would be enrolled into the index cohort. Depending on the trial arm they were assigned to, women were provided with materials and brief training for their male partner, assuming only one probable father. We included women who were attending their first ANC visit; aged at least 18 years; intending to remain in the clinic catchment area; not already enrolled; and whose male partner (ie, the child's probable father) was present and reachable, not already receiving ART, or not already tested during this pregnancy. We excluded women if their male partner was aged younger than 18 years, already receiving ART, and not presently available and resident in the catchment area.

People newly diagnosed with HIV during routine clinic HIV testing, including pregnant women, were enrolled into the index cohort. We included individuals who were aged at least 18 years, intending to remain in the clinic catchment area, not already enrolled, and had at least one sexual contact (ie, contactable sexual partner) not already known to be HIV-positive. We excluded index patients if all of their sexual contacts were aged younger than 18 years or were already receiving ART, or if none were presently available and resident in the catchment area. Depending on the trial arm they were assigned to, index patients were provided with materials and brief training, and asked to deliver materials to all sexual contacts over the past 12 months. The number of sexual contacts per index patient varied on the basis of the number of contactable sexual partners.

### Randomisation and masking

Restricted randomisation (blocking) was used to randomise the 27 clusters to three arms in a ratio of 1:1:1, with district and HIV prevalence in ANC as the variables for restriction. The first group was enhanced standard of care (standard care group), which only offered letters inviting male partners or sexual contacts to attend HIV testing services at the clinic. The second group was HIVST only (HIVST only group), which offered invitation letters plus oral HIVST kits to the pregnant women to deliver to their male partners or to the index patients to deliver to their sexual contacts. The third group was HIVST with an additional financial incentive (HIVST plus financial incentive group), which offered male partners or sexual contacts US$10 to retest at the clinic following self-testing, irrespective of HIV status. A statistician (MN) generated a list of 100 000 randomisation options meeting the restriction criteria and chose one of these options at random using a computer programme. At a public ceremony held on June 29, 2018, representatives of the 27 primary health centres and hospitals were split into three groups according to the letters of their randomisation allocation. Each group chose a team name and a team captain. The team captains drew a golf ball at random, which was prelabelled with the trial arm to be implemented at the nine clinics within their group.

Due to the nature of the interventions, clinic personnel and participants could not be masked to allocation; however, data were managed by an appointed data manager without reference to the trial arm to maintain investigator masking until final analysis.

### Procedures

In the standard care group, letters (ie, standard family referral slips) were used to invite male partners of women in the ANC cohort or sexual partners of patients in the index cohort to attend HIV testing services within 28 days of enrolment. The HIVST only group used the approach intended for routine adoption in Malawi, with only HIV-positive partners advised to return for confirmation and HIV care. The HIVST plus financial incentive group allowed the diagnostic accuracy of HIVST kits distributed in this way to be investigated. The participant's own interpretation of HIVST results and results obtained from retesting by the HIV provider were compared. This group was also offered voluntary male medical circumcision (VMMC) for uncircumcised men who were HIV-negative.

In the HIVST only group, women in the ANC cohort or patients in the index cohort were provided with one OraQuick HIV Self-Test (OraSure Technologies, Bethlehem, PA, USA) kit per sexual partner for secondary distribution, with a brief demonstration (approximately 5 min) on how to correctly open the kit, identify the instructions for use, run the test, and read the results. Partners were encouraged to attend clinic services to confirm all positive results or results that were serodiscordant (ie, one positive and one negative) with their regular partner.

In the HIVST plus financial incentive group, women in the ANC cohort or patients in the index cohort were, in addition to the test kit and demonstration, provided with a leaflet offering their male partner or sexual contacts $10 to retest at the clinic. Demand-side financial incentives (ie, incentives offered to users of health services) have become of interest in HIV, especially for increasing the uptake of testing and clinic attendance in priority populations.^14^

Government staff managed secondary HIVST kit distribution and HIV care, including confirmatory testing and starting patients on ART. Follow-up interviews with women in the ANC cohort were completed by an interviewer, independent from the original kit distributor, at the next ANC visit (within 28 days) to ascertain male partner testing. There was no compensation for completing this interview.

Follow-up testing was done by accredited providers using two fingerprick rapid diagnostic tests in parallel (Determine [Abbott; Chicago, IL, USA] and Uni-Gold [Trinity Biotech; Bray, Ireland]) for all male partners or sexual contacts who collected their $10 incentive and reported having self-tested in the HIVST plus financial incentive group. HIVST results were self-reported, with inspection of used HIVST kits if brought by the client. Participants had to show a used HIVST kit; if the used HIVST was not available, the participant was asked to do a second HIVST before follow-up testing.

### Outcomes

The primary outcome for the ANC cohort was the proportion of male partners reportedly tested for HIV, as ascertained by interview with the woman attending ANC 28 days after enrolment. The primary outcome for the index cohort was the geometric mean number of HIV-positive people diagnosed per cluster, within 28 days of enrolling the index patient. To satisfy the primary outcome, within 28 days of enrolling the index patient, a sexual contact of a participant in the standard care group had to present the originally allocated letter to the clinic and test positive, and a sexual contact of a participant in the HIVST only group and HIVST plus financial incentive group had to bring a positive self-test result to be immediately confirmed.

Secondary outcomes for the ANC cohort were the proportion of male partners attending any HIV clinic services, or who started ART, underwent VMMC, or attended a discordant couples' clinic within 28 days of enrolling participants, using all eligible women as the denominator. A post-hoc analysis for this cohort was the proportion of male partners booked for VMMC, also using all eligible women as the denominator.

Secondary outcomes for the index cohort were the proportion of sexual contacts reportedly tested for HIV, as ascertained by interview with the index patient at day 28; the number of sexual contacts who attended the clinic for any HIV services; and the number of sexual contacts who started ART, underwent VMMC, or attended a discordant couples' clinic, within 28 days of the index patient's diagnosis. A secondary outcome for both cohorts was the percentage of adverse events (eg, partnership breakdown, intimate partner violence, etc) related to HIVST reported by participants, measured at day 28.

Outcomes were evaluated at day 28 by use of participant interviews and clinic registers, including phone calls for clients with missed interviews.

We also did a prespecified exploratory analysis of secondary accuracy of HIVST—the correctness (ie, sensitivity and specificity) with which the ultimate user (not necessarily the original recipient of the test kit) does the test—in the HIVST plus financial incentive group.

### Statistical analysis

The sample size calculation was based on the primary outcome of the ANC cohort, with nine clusters per arm and 350 pregnant women per cluster, providing 90% power to detect a 12% increase in partner HIV testing over an assumed 20% in the standard care group, using a coefficient of variation (*k*) of 0·25.^4^

ANC cohort analyses followed intention-to-treat principles, so all eligible women (not just those who participated) were included in the denominator.^16^ A per-protocol post-hoc analysis^16^ was also done for the ANC cohort, excluding women not interviewed at follow-up. The main hypothesis testing in the ANC cohort used cluster-level summaries and a *t* test due to the small number of clusters per arm, with methods appropriate for clustered trial designs.^17^ In summary, the proportion achieving the primary outcome was computed for each cluster. The mean of proportions in each intervention arm was then compared with the mean of proportions in the standard care group with a *t* test. Risk ratios (RRs), risk differences, and 95% CIs were computed for male partner testing. A sensitivity analysis using random-effects logistic regression was done. For the primary outcome of the index cohort, analysis followed intention-to-treat principles. The geometric mean count of HIV-positive contacts was calculated, with a negative binomial model used for incidence rate ratios (IRR) comparing each intervention group with the standard care group, to account for overdispersion.

Analyses for the secondary outcomes followed intention-to-treat principles, with all male partners or sexual contacts achieving the outcome in the numerator and all eligible women in the ANC cohort or eligible patients in the index cohort in the denominator.

As a sensitivity analysis, an individual-level, random-effects logistic model was fitted for the ANC cohort to explore any potential differences from the main analysis.

To estimate secondary accuracy, the sensitivity and specificity and associated 95% CIs of participants' own interpretation of self-test results were computed by use of HIV provider-administered parallel rapid testing, with Uni-Gold (Abbott) and Determine (Biotech) as the reference.

Statistical analysis was done with R (version 5.1) and Stata (14.0).

This trial is registered with ClinicalTrials.gov, NCT03705611.

### Role of the funding source

The funder of the study had no role in study design, data collection, data analysis, data interpretation, or writing of the report.

## Results

We completed this study between Sept 8, 2018, and May 2, 2019. In the ANC cohort, a total of 9939 women were assessed for eligibility, of whom 4544 (45·7%) were eligible (figure). 5395 (54·3%) women were excluded largely because they were accompanied by the male partner to the ANC visit. Nine clusters were randomly assigned to each group, resulting in 1447 (31·8%) of 4544 women in the standard care group, 1465 (32·2%) in the HIVST only group, and 1632 (35·9%) in the HIVST plus financial incentive group. 83 (1·8%) of 4544 eligible women did not consent to participate and were not recruited, but were still included in the analysis population: 51 (3·5%) of 1447 women in the standard of care group, 15 (1·0%) of 1465 in the HIVST only group, and 17 (1·0%) of 1632 in the HIVST plus financial incentive group. Of the 4461 women recruited, 315 (22·6%) of 1396 individuals in the standard care group, 285 (17·6%) of 1615 individuals in the HIVST only group, and 483 (33·3%) of 1450 individuals in the HIVST plus financial incentive group were lost to follow-up at 28 days. No cluster was lost to follow-up; hence, all clusters were included in the analysis and all eligible women were included in the analysis of the primary outcome.

**Figure:**
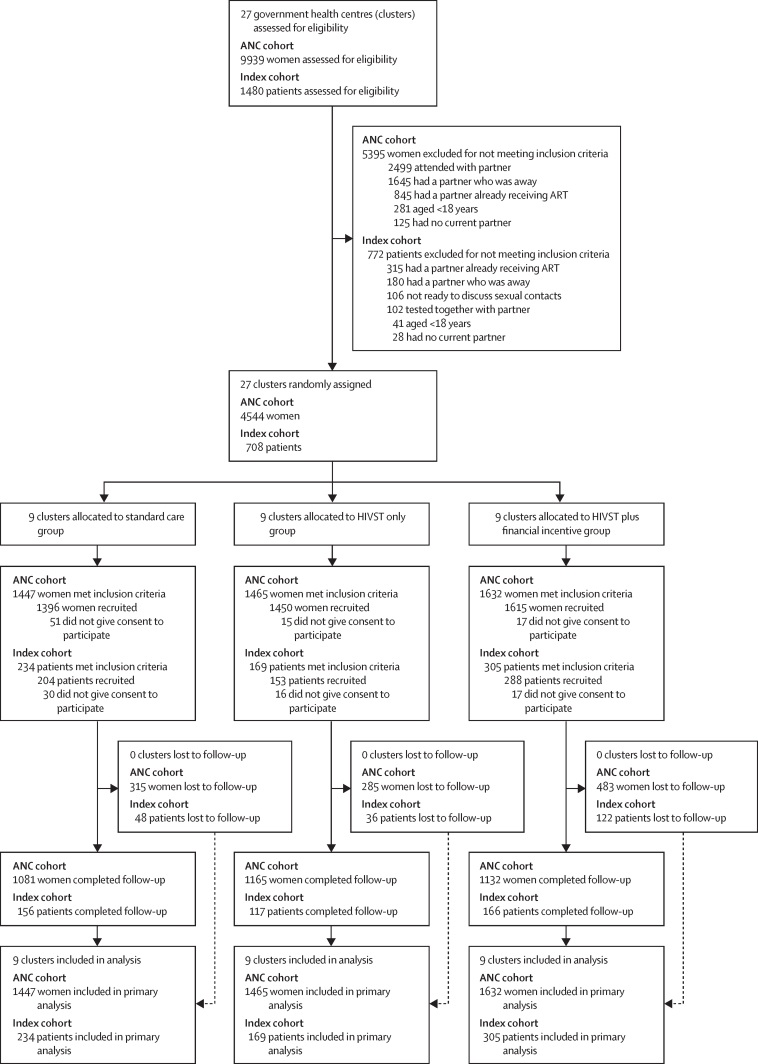
Trial profile ANC=antenatal care. ART=antiretroviral therapy. HIVST=HIV self-testing.

In the index cohort, a total of 1480 index patients were assessed for eligibility, of whom 708 (47·8%) were eligible. We excluded 772 (52·2%) patients mostly due to their sexual contact already receiving ART. Nine clusters were randomly assigned to each group, resulting in 234 (33·1%) patients in the standard care group, 169 (23·9%) in the HIVST only group, and 305 (42·9%) in the HIVST plus financial incentive group. 63 (8·9%) of 708 eligible patients did not consent to participate and were not recruited, but were still included in the analysis population: 30 (12·8%) of 234 patients in the standard of care group, 16 (9·5%) of 169 in the HIVST only group, and 17 (5·6%) of 305 in the HIVST plus financial incentive group. Among the 645 index patients recruited, 48 (23·5%) of 204 individuals in the standard care group, 36 (23·5%) of 153 individuals in the HIVST only group, and 122 (42·4%) of 288 individuals in the HIVST plus financial incentive group were lost to follow-up at 28 days. No clusters were lost to follow-up and were all included in the analysis, with all eligible index patients included in the analysis of the primary outcome.

Baseline characteristics of women in the ANC cohort were reasonably balanced (table 1), except for illiteracy (247 [17·1%] of 1447 in the standard care group *vs* 95 [6·5%] of 1465 in the HIVST only and 104 [6·4%] of 1632 in the HIVST plus financial incentive group) and reported previous partner HIV testing more or less than 12 months ago (516 [35·7%] of 1447 in the standard care group *vs* 651 [44·4%] of 1465 in the HIVST only group and 899 [55·1%] of 1632 in the HIVST plus financial incentive group).

**Table 1:** Characteristics of women in the ANC cohort and their male partners, as reported by these women at enrolment

			**Standard care group (n=1447)**	**HIVST only group (n=1465)**	**HIVST plus financial incentive group (n=1632)**
Number of clusters			9	9	9
Harmonic mean number per cluster			90	138	136
Women in the ANC cohort					
	Age, years		22 (19–28)	22 (19–27)	23 (19–33)
	Able to read and write				
		Yes	1176 (81·3%)	1368 (93·4%)	1524 (93·4%)
		No	247 (17·1%)	95 (6·5%)	104 (6·4%)
		Data missing	24 (1·6%)	2 (0·1%)	4 (0·2%)
	HIV test results before trial				
		Positive	16 (1·1%)	11 (0·8%)	13 (0·8%)
		Negative	1415 (97·8%)	1452 (99·1%)	1617 (99·1%)
		Data missing	16 (1·1%)	2 (0·1%)	2 (0·1%)
	Intimate partner violence or adverse behaviour, or marital separation, in past 12 months				
		Physical violence	23 (1·6%)	6 (0·4%)	39 (2·4%)
		Forced sex	0	1 (0·1%)	4 (0·3%)
		Threatened	6 (0·4%)	3 (0·2%)	9 (0·5%)
		Shouted at	33 (2·3%)	6 (0·4%)	59 (3·6%)
		Ignored	14 (1·0%)	3 (0·2%)	10 (0·6%)
		Marital separation	18 (1·2%)	3 (0·2%)	9 (0·5%)
		Denied household needs	3 (0·2%)	0	6 (0·4%)
		None	1280 (88·5%)	1434 (97·9%)	1490 (91·3%)
		Data missing	70 (4·8%)	9 (0·6%)	6 (0·4%)
Male partners					
	Age, years		26 (22–32)	26 (22–33)	27 (23–33)
	HIV testing history				
		Never tested before	409 (28·3%)	520 (35·6%)	422 (25·7%)
		Tested >12 months ago	253 (17·6%)	443 (30·2%)	343 (21·1%)
		Tested ≤12 months ago	263 (18·1%)	208 (14·2%)	556 (34·1%)
		Unsure	345 (23·8%)	248 (16·7%)	245 (15·0%)
		Data missing	177 (12·2%)	46 (3·1%)	66 (4·1%)

Data are median (IQR) or n (%). HIVST=HIV self-testing. ANC=antenatal care.

For the index cohort, there was reasonable balance in baseline characteristics (table 2), although there were more men in the HIVST plus financial incentive group than in the HIVST only and standard care groups (99 [32·5%] of 305 *vs* 58 [24·8%] of 234 in the standard care group and 36 [21·3%] of 169 in the HIVST only group), and higher illiteracy in the standard care group (59 [25·2%] of 234) than in the HIVST only (22 [13·0%] of 169) and HIVST plus financial incentive (27 [8·8%] of 305) groups.

**Table 2:** Characteristics of patients in the index cohort and their sexual contacts, as reported by index patients at enrolment

			**Standard care group (n=234)**	**HIVST only group (n=169)**	**HIVST plus financial incentive group (n=305)**
Number of clusters			9	9	9
Harmonic mean number per cluster			90	138	136
Patients in the index cohort					
	Sex				
		Male	58 (24·8%)	36 (21·3%)	99 (32·5%)
		Female	175 (74·8%)	132 (78·1%)	206 (67·5%)
		Data missing	1 (0·4%)	1 (0·6%)	0
	Age, years		27 (23–35)	28 (22–34)	30 (24–36)
	Able to read and write				
		Yes	166 (70·9%)	144 (85·2%)	272 (89·2%)
		No	59 (25·2%)	22 (13·0%)	27 (8·8%)
		Data missing	9 (3·9%)	3 (1·8%)	6 (2·0%)
	Intimate partner violence or adverse behaviour, or marital separation, in past 12 months				
		Physical violence	2 (0·9%)	1 (0·6%)	6 (2·0%)
		Forced sex	0	0	1 (0·3%)
		Threatened	1 (0·4%)	0	0
		Shouted at	7 (3·0%)	1 (0·6%)	16 (5·3%)
		Ignored	5 (2·1%)	0	4 (1·3%)
		Marital separation	9 (3·9%)	1 (0·6%)	5 (1·6%)
		Denied household needs	0	0	1 (0·3%)
		None	199 (85·0%)	161 (95·3%)	259 (84·9%)
		Data missing	11 (4·7%)	5 (3·0%)	13 (4·3%)
	Number of sexual contacts in past 12 months				
		1	198 (84·6%)	153 (90·5%)	269 (88·2%)
		2–4	24 (10·3%)	9 (5·3%)	32 (10·5%)
		Data missing	12 (5·1%)	7 (4·2%)	4 (1·3%)
	Number of sexual contacts still in contact with				
		1	209 (89·3%)	162 (95·8%)	284 (93·1%)
		2–4	10 (4·3%)	3 (1·8%)	17 (5·6%)
		Data missing	15 (6·4%)	4 (2·4%)	4 (1·3%)
	Number of sexual contacts able to give letter or kits				
		1	209 (89·3%)	155 (91·7%)	285 (93·4%)
		2–4	8 (3·4%)	3 (1·8%)	16 (5·3%)
		Data missing	17 (7·3%)	11 (6·5%)	4 (1·3%)
Sexual contacts					
	Sex				
		Male	169 (72·2%)	132 (78·1%)	212 (69·5%)
		Female	60 (25·6%)	34 (20·1%)	92 (30·2%)
		Data missing	5 (2·2%)	3 (1·8%)	1 (0·3%)
	Age, years		30 (26–37)	30 (25–37)	32 (26–37)
	Testing together for HIV				
		Never tested before	124 (53·0%)	96 (56·8%)	161 (52·8%)
		Tested >12 months ago	62 (26·5%)	33 (19·5%)	94 (30·8%)
		Tested ≤12 months ago	37 (15·8%)	36 (21·3%)	47 (15·4%)
		Data missing	11 (4·7%)	4 (2·4%)	3 (1·0%)
	HIV status				
		Positive	10 (4·3%)	26 (15·4%)	12 (3·9%)
		Negative	62 (26·5%)	19 (11·2%)	83 (27·2%)
		Unknown	157 (67·1%)	121 (71·6%)	207 (67·9%)
		Data missing	5 (2·1%)	3 (1·8%)	3 (1·0%)

Data are median (IQR) or n (%). HIVST=HIV self-testing.

For the primary outcome in the ANC cohort, mean uptake per cluster of male partner HIV testing by 28 days, as reported by women in this cohort, was 73·0% (SD 13·1) in the HIVST only group and 65·2% (11·6) in the HIVST plus financial incentive group, compared with 35·0% (10·0) in the standard care group (table 3). In comparison with the standard care group, the adjusted RR was 1·71 (95% CI 1·48–1·98, p<0·0001) for the HIVST only and 1·62 (1·45–1·81, p<0·0001) for the HIVST plus financial incentive groups (table 3; appendix p 1). The coefficient of variation (*k*) for the male partner testing outcome was 0·15.

**Table 3:** Reported testing of male partners of women in the ANC cohort and newly diagnosed HIV-positive people among sexual contacts of patients in the index cohort

			**Standard care group**	**HIVST only group**	**HIVST plus financial incentive group**
**Women in the ANC cohort and their male partners**					
Number of eligible women			1447	1465	1632
Number of male partners tested*			498 (34·4%)	1106 (75·5%)	1000 (61·3%)
	Mean proportion per cluster, % (SD)		35·0% (10·0)	73·0% (13·1)	65·2% (11·6)
	p value		..	<0·0001	<0·0001
	Risk difference (95% CI) *vs* standard care		..	38·3% (35·0–41·7)	30·1% (26·8–33·5)
	Unadjusted RR (95% CI)		1 (ref)	2·08 (1·64–2·64)	1·86 (1·47–2·35)
		p value	..	<0·0001	<0·0001
	Adjusted RR† (95% CI)		1 (ref)	1·71 (1·48–1·98)	1·62 (1·45–1·81)
		p value	..	<0·0001	<0·0001
**Per-protocol analysis of women in the ANC cohort and their male partners**					
Number of women interviewed at follow-up			1081	1165	1132
Number of male partners tested*			498 (46·1%)	1106 (94·9%)	1000 (88·3%)
	Mean proportion per cluster, %		48·7%	93·7%	89·1%
	Risk difference (95% CI) *vs* standard care		..	48·9% (41·2–56·5)	42·3% (33·7–50·8)
	Unadjusted RR (95% CI)		1 (ref)	3·09 (2·52–3·65)	2·18 (1·66–2·71)
		p value	..	<0·0001	<0·0001
	Adjusted RR† (95% CI)		1 (ref)	3·04 (2·38–3·70)	2·07 (1·49–2·66)
		p value	..	<0·0001	<0·0001
**Patients in the index cohort and their sexual contacts**					
Number of eligible index patients			234	169	305
Number of sexual contacts reached by index patients			209	155	285
Number of sexual contacts testing HIV-positive‡			9/209 (4·3%)	13/155 (8·4%)	32/285 (11·2%)§
	Geometric mean per cluster (SD)		1·35 (1·62)	1·91 (1·78)	3·20 (3·81)
	Risk difference (95% CI) *vs* standard care		..	4·3% (2·2–6·4)	6·9% (3·2–9·1)
	Unadjusted incidence RR (95% CI)		1 (ref)	1·38 (0·45–4·16)	3·24 (1·16–9·07)
		p value	..	0·5660	0·0250
	Adjusted incidence RR¶ (95% CI)		1 (ref)	1·65 (0·49–5·55)	3·11 (0·99–9·77)
		p value	..	0·3370	0·0440

Each trial arm had nine clusters. HIVST=HIV self-testing. ANC=antenatal care. RR=risk ratio.

* Measured within 28 days, as reported by women in the ANC cohort at their next ANC visit (*k*=0·15).

† Adjusted for woman's literacy and male partner's HIV testing history.

‡ Extracted from clinic records and confirmed to be newly diagnosed as HIV-positive (*k*=0·75).

§ 19 individuals from Mulanje District Hospital (Mulanje) and nine from Mbayani Primary Health Centre (Blantyre).

¶ Adjusted for the number eligible as an offset in the negative binomial model.

For the primary outcome in the index cohort, the geometric mean number of new positive sexual partners per cluster was 1·35 (SD 1·62) for the standard care group, 1·91 (1·78) for the HIVST only group, and 3·20 (3·81) for the HIVST plus financial incentive group (table 3; appendix p 2). The HIVST plus financial incentive group had a higher incidence of newly diagnosed HIV-positive sexual contacts than did the standard care group (IRR 3·24, 95% CI 1·16–9·07; p=0·0250); however, there was no difference in rates between the HIVST only group and the standard care group (IRR 1·38, 0·45–4·16; p=0·5660). In an analysis adjusted for number of eligible index patients per cluster (which was more highly variable or overdispersed than anticipated in our statistical analysis plan), the evidence for difference with the HIVST plus financial incentive intervention was weaker, with an adjusted IRR of 3·11 (0·99–9·77; p=0·0440). The coefficient of variation (*k*) for the male partner testing outcome was 0·75.

Secondary outcomes in the ANC cohort should be interpreted in light of the different instructions given per arm—ie, all partners were asked to attend clinic HIV services in the standard care and HIVST plus financial incentive groups, yet only men testing positive with HIVST and serodiscordant couples were asked to attend HIV services in the HIVST only group. Thus, the main comparison for the proportion of male partners attending any HIV clinic services was the standard care group versus the HIVST plus financial incentive group (table 4). Providing HIVST plus $10 for clinic attendance substantially increased male partner attendance, from a mean of 19·7% (SD 7·6) per cluster in the standard care group to 76·5% (20·0) in the HIVST plus financial incentive group (adjusted RR 2·84, 95% CI 2·37–3·41; table 4). The proportion of male partners starting ART, undergoing VMMC, or attending discordant couples' clinic was 168 (11·6%) of 1447 eligible women in the standard care group versus 309 (18·9%) of 1632 eligible women in the HIVST plus financial incentive group (p<0·0001; appendix p 3).

**Table 4:** Clinic attendance and VMMC among male partners of women in the ANC cohort and HIV testing among sexual contacts of patients in the index cohort

			**Standard care group**	**HIVST only group**	**HIVST plus financial incentive group**
**Women in the ANC cohort and their male partners**					
Number of eligible women			1447	1465	1632
Number of male partners seen at the clinic*			228 (15·8%)	NA*	1232 (75·5%)
	Mean proportion per cluster, % (SD)		19·7% (7·6)	NA	76·5% (20·0)
		p value	..	NA	<0·0001
	Risk difference (95% CI) *vs* standard care		..	NA	56·8% (53·7 to 59·5)
	Unadjusted RR (95% CI)		1 (ref)	NA	4·51 (3·10 to 6·55)
		p value	..	NA	<0·0001
	Adjusted RR (95% CI)†		1 (ref)	NA	2·84 (2·37 to 3·41)
		p value	..	NA	<0·0001
Number of men circumcised‡			3 (0·2%)	NA	21 (1·3%)
Number of men who started ART			2 (0·1%)	0	22 (1·5%)
Number of men who attended a discordant couples' clinic			NM	NM	NM
Number of newly diagnosed HIV-positive partners seen at the clinic			2 (0·1%)	0	22 (1·5%)
Number of men booked for VMMC			133 (9·2%)	NA	211 (12·9%)
	Risk difference (95% CI) *vs* standard care		..	NA	3·7% (−6·6 to 24·4)
**Patients in the index cohort and their sexual contacts**					
Number of eligible index patients			234	169	305
Number of sexual contacts tested for HIV§			82 (35·0%)	101 (59·8%)	128 (42·0%)
	Mean proportion per cluster, %		34·3%	49·7%	51·7%
	Risk difference (95% CI) *vs* standard care		..	15·4% (5·9 to 25·4)	17·4% (9·7 to 25·5)
	Unadjusted RR (95% CI)		1 (ref)	1·57 (1·34 to 1·85)	1·45 (1·23 to 1·70)
		p value	..	<0·0001	<0·0001
	Adjusted RR (95% CI)†		1 (ref)	1·57 (1·22 to 2·02)	1·45 (1·17 to 1·79)
		p value	..	<0·0001	<0·0001

Each trial arm had nine clusters. HIVST=HIV self-testing. ANC=antenatal care. RR=risk ratio. NA=not applicable. NM=not measured. VMMC=voluntary male medical circumcision.

* Measured by clinic attendance with a pre-allocated barcoded card. Not measured in the HIVST only group because only individuals with a positive HIVST were encouraged to attend the clinic, implying uncertainty in the denominator.

† Adjusted for clustering.

‡ Using number of men eligible for VMMC as the denominator (in part reflects supply-side barriers in accessing VMMC in Malawi).

§ As reported by the index client at their antiretroviral therapy refill visit.

For the secondary outcomes in the index cohort, the mean proportion per cluster of eligible sexual partners reported by the index patients as having tested for HIV within 28 days was higher in the HIVST only group and in the HIVST plus financial incentive group than in the standard care group (table 4). The number of sexual contacts attending the clinic for any HIV services was 51 (21·8%) of 234 eligible patients in the standard care group, 17 (10·1%) of 169 in the HIVST only group, and 181 (59·3%) of 305 in the HIVST plus financial incentive group. The number of sexual contacts who started ART, underwent VMMC, or attended discordant couples' clinic was 60 (19·7%) of 305 individuals in the HIVST plus financial incentive group as per intention-to-treat analysis, compared with 12 (5·1%) of 234 in the standard care group and 16 (9·5%) of 169 in the HIVST only group.

Only four (0·1%) of the 5252 individuals combined reported a marital separation related to self-testing at the day 28 interview; two in the HIVST only group and two in the HIVST plus financial incentive group, both of which were from the ANC cohort. No other adverse events were reported.

In the ANC cohort, differences in the primary outcome between each trial arm were more pronounced in the per-protocol analysis (table 3). The highest reported HIV testing coverage was in the HIVST only group. Results from an individual-level, random-effects logistic model were similar to those obtained from the cluster-level summaries approach for the ANC cohort primary outcome of male partner testing within 28 days (data not shown).

Among 863 sexual partners retested in the HIVST plus financial incentive group of both cohorts, HIVST sensitivity was 89·7% (95% CI 78·8–96·1) with 52 of 58 partners with HIV correctly identified through HIVST, and specificity was 99·5% (98·7–99·9), with 801 of 805 partners correctly identified as not having HIV through HIVST. Of the 52 true-positive patients newly diagnosed by HIVST, 20 were partners of HIV-negative women attending ANC and 32 were sexual contacts of index patients. There were an additional six false-negative, four false-positive, and 807 true-negative patients. All four false-positive results reflected misinterpretation of the kits showing negative results, whereas the six false-negative results were in patients with undetectable viral loads (<1000 copies per mL of blood).

In post-hoc analyses, we found that the proportion of male partners booked for VMMC among HIV-negative men (appendix p 3) and the proportion of male partners with new HIV diagnoses (table 4) were numerically higher in the HIVST plus financial incentive group than in the standard care group; however, the proportion of male partners booked for VMMC among all eligible women did not substantially differ between these two groups (table 4).

## Discussion

This pragmatic, cluster-randomised trial showed that routinely offering oral HIVST kits through secondary distribution could be readily integrated into routine clinical practice in rural health centres and district hospitals, with high participation and minimal harms. Secondary distribution provided a highly acceptable out-of-clinic strategy that substantially increased HIV testing by male partners of pregnant women, confirming high potential to contribute to elimination of mother-to-child transmission targets. In this first trial of HIVST for index testing, secondary distribution of kits from HIV testing clinics reached half (228 [48·1%]) of all 474 reported sexual contacts of people with newly diagnosed HIV. Study power was limited by low prevalence of undiagnosed HIV in Malawi and high clinic-level variability. However, additional research is needed to maximise accuracy from kits distributed by partners.

Secondary distribution of HIVST kits for pregnant women has consistently outperformed alternative approaches with regard to increasing HIV testing, and should now be considered regional best practice.^4^, ^5^, ^6^ HIV incidence during pregnancy and breastfeeding is well above rates in women who are not pregnant,^18^ making male partner testing and intensified HIV prevention a crucial part of strategies to eliminate mother-to-child transmission of HIV.^1^ To maximise impact from strategies based on HIVST, national programmes should invest in operational research to monitor and optimise the accuracy of secondary distribution of HIVST, and to create demand for proven serostatus-based HIV prevention approaches by the male partner, notably prompt ART if HIV-positive^19^, ^20^ and VMMC if HIV-negative.^21^, ^22^, ^23^

In this trial, we showed that a conditional financial incentive ($10) to retest after HIVST can stimulate prompt confirmatory testing, as well as engagement with VMMC services in Malawi.^4^, ^24^, ^25^, ^26^ Nevertheless, incentives are prone to unintended consequences and implementation challenges, and might undermine sustainability and universal health coverage initiatives.^1^ Some women are worried about men using their incentive to buy alcohol or transactional sex.^11^ Failure to pass on kits because of this concern might explain the less complete follow-up for women from financial incentive clinics (1132 [70·1%] of 1615 women recruited) compared with women from non-incentivised clinics (2246 [78·9%] of 2846), with more pronounced differences for the index cohort. Use of non-cash incentives might overcome immediate misuse of cash incentives. Drawing on experiences of disbursing non-cash humanitarian aid and subsidies by governments in resource-limited settings can overcome logistical challenges in implementing incentive-based HIV programmes.

This was the first trial of HIVST to enable contact tracing and testing for clinic attendees with newly diagnosed HIV (ie, index testing). Kits were reportedly used by half (229 [48·3%] of 474) of the contacts in the index cohort without requiring costly and labour-intensive home visits.^9^, ^27^ However, this finding might be an underestimate because our analysis assumes no uptake by partners of patients who were not re-interviewed. Index testing is feasible and retains high yield, even in settings with low HIV prevalence.^9^, ^27^

Partners who received the HIVST kit via secondary distribution appeared to have some challenges and had lower sensitivity (89·7%: 52 true-positive patients and six false-negative patients) than did trained providers and individuals in previous studies, including those who directly received a kit.^28^ This difference highlights the importance of ensuring that secondary distribution of HIVST is coupled with sufficient information, such as shareable videoclips or other materials, aiming to improve this situation by promoting consistently correct kit use and supporting interpretation.^29^

Consistent with previous studies, only four self-resolving temporary marital separations occurred (equating to 0·1% of all participants), with no other adverse events reported.^4^, ^7^

This study had several strengths and novel aspects. First, the trial had a cluster-randomised design and was large scale, with 27 government clinics as the unit of randomisation (including a previously unstudied priority patient cohort [index patients]) and ANC clinic attendees. This design is key and differs substantially from previous studies that were relatively small^4^ and individually randomised,^5^, ^30^, ^31^ or cohort studies,^6^, ^32^ which have a huge potential for contamination and bias. Second, the interventions were delivered via a pragmatic design by Ministry of Health personnel, as would be the case during routine implementation (including kit supply chain, distribution, and documentation). Third, secondary accuracy was investigated, which is a key assumption in any secondary distribution study or programme. The idea with secondary distribution is that the kit is offered indirectly to someone who is not present, through a proxy. Until our study, to our knowledge, we were not aware of any large study that explored secondary accuracy with HIVST kits delivered via antenatal care attendees or index patients. Finally, financial incentives were used to investigate clinic attendance following receipt of HIVST kits by male partners of women in the ANC cohort and by sexual contacts of patients in the index cohort.

Limitations of this study include statistical power for the index cohort outcomes, with Malawi being close to HIV elimination targets. Notably, variability between clusters was extremely high for the index cohort primary outcome of numbers of newly diagnosed HIV-positive contacts per clinic (*k*=0·75). Although we adapted our pre-planned analysis to use negative binomial instead of Poisson regression, there might still have been residual overdispersion affecting confidence intervals. However, a probable true effect is supported by the important impact of the HIVST plus financial incentive group on male partner attendance from ANC clinics. The primary ANC cohort outcome (reported male partner testing) and equivalent index cohort secondary outcomes were based on interview with the kit distributor, not the recipient, and are, therefore, prone to reporting bias. There is also potential for systematic errors from the rapid HIV test kits used and for misclassification of HIVST results due to user-reading errors. Furthermore, VMMC outcomes in Malawi are limited by supply side issues, requiring a minimum of ten men to trigger outreach arrangements with a private provider; therefore, only a small proportion of men expressing interest in VMMC had surgery.^4^

Strategies that are better targeted and more efficient at testing are needed for HIV programmes to carefully scale back intensity without jeopardising programmatic gains.^1^, ^10^ We have shown that secondary distribution of HIVST kits can be readily integrated into routine clinic activities, with high uptake by intended users and few adverse events. Secondary distribution of HIVST kits for pregnant women should now be considered regional priority.^4^, ^5^, ^6^ Optimising HIVST accuracy, especially in economically disadvantaged rural communities, remains an important programmatic challenge.^29^ In any case, novel strategies for maintaining HIV services are needed during the COVID-19 pandemic, and HIVST-based approaches have the major advantage of not requiring direct interaction between contacts and providers until confirmatory testing and enrolment into HIV care. HIVST can meet unique programmatic needs and global HIV testing targets, both during the COVID-19 pandemic and in the long term, and should be implemented routinely in all settings with high HIV prevalence.

## Data sharing

Individual de-identified participant data (including data dictionaries) will be shared including baseline data and primary and secondary outcomes data via the London School of Hygiene & Tropical Medicine Data Compass. The data will become permanently available upon publication of the manuscript. The data will be openly accessible to anyone who wishes to perform any analysis. A link to the data will be provided.

## Declaration of interests

ELC reports grants from the London School of Hygiene & Tropical Medicine, during the conduct of the study. CCJ reports grants from Unitaid, during the conduct of the study; and grants from Bill & Melinda Gates Foundation and the US Agency for International Development, outside of the submitted work. All other authors declare no competing interests.

## References

[1] WHO (2019). Consolidated guidelines on HIV testing services for a changing epidemic: policy brief.

[2] WHO (2016). Guidelines on HIV self-testing and partner notification. Supplement to consolidated guidelines on HIV testing services.

[3] UNAIDS (2020). Global AIDS Monitoring.

[4] Choko AT, Corbett EL, Stallard N (2019). HIV self-testing alone or with additional interventions, including financial incentives, and linkage to care or prevention among male partners of antenatal care clinic attendees in Malawi: an adaptive multi-arm, multi-stage cluster randomised trial. PLoS Med.

[5] Masters SH, Agot K, Obonyo B, Napierala Mavedzenge S, Maman S, Thirumurthy H (2016). Promoting partner testing and couples testing through secondary distribution of HIV self-tests: a randomized clinical trial. PLoS Med.

[6] Thirumurthy H, Masters SH, Mavedzenge SN, Maman S, Omanga E, Agot K (2016). Promoting male partner HIV testing and safer sexual decision making through secondary distribution of self-tests by HIV-negative female sex workers and women receiving antenatal and post-partum care in Kenya: a cohort study. Lancet HIV.

[7] Kumwenda MK, Johnson CC, Choko AT (2019). Exploring social harms during distribution of HIV self-testing kits using mixed-methods approaches in Malawi. J Int AIDS Soc.

[8] Drake AL, Wagner A, Richardson B, John-Stewart G (2014). Incident HIV during pregnancy and postpartum and risk of mother-to-child HIV transmission: a systematic review and meta-analysis. PLoS Med.

[9] Lasry A, Medley A, Behel S (2019). scaling up testing for human immunodeficiency virus infection among contacts of index patients–20 countries, 2016–2018. MMWR Morb Mortal Wkly Rep.

[10] UNAIDS (2019). UNAIDS data 2019.

[11] Choko AT, Kumwenda MK, Johnson CC (2017). Acceptability of woman-delivered HIV self-testing to the male partner, and additional interventions: a qualitative study of antenatal care participants in Malawi. J Int AIDS Soc.

[12] Tembo TA, Kim MH, Simon KR (2019). Enhancing an HIV index case testing passive referral model through a behavioural skills-building training for healthcare providers: a pre-/post-assessment in Mangochi District, Malawi. J Int AIDS Soc.

[13] Sakala D, Kumwenda MK, Conserve DF, Ebenso B, Choko AT (2021). Socio-cultural and economic barriers, and facilitators influencing men's involvement in antenatal care including HIV testing: a qualitative study from urban Blantyre, Malawi. BMC Public Health.

[14] Choko AT, Candfield S, Maheswaran H, Lepine A, Corbett EL, Fielding K (2018). The effect of demand-side financial incentives for increasing linkage into HIV treatment and voluntary medical male circumcision: a systematic review and meta-analysis of randomised controlled trials in low- and middle-income countries. PLoS One.

[15] Youngs J, Hooper C (2015). Ethical implications of HIV self-testing. J Med Ethics.

[16] Gupta SK (2011). Intention-to-treat concept: a review. Perspect Clin Res.

[17] Hayes R, Moulton LH (2009). Cluster randomised trials.

[18] Chi BH, Rosenberg NE, Mweemba O (2018). Involving both parents in HIV prevention during pregnancy and breastfeeding. Bull World Health Organ.

[19] Lundgren JD, Babiker AG, Gordin F (2015). Initiation of antiretroviral therapy in early asymptomatic HIV infection. N Engl J Med.

[20] Hayes RJ, Donnell D, Floyd S (2019). Effect of universal testing and treatment on HIV incidence - HPTN 071 (PopART). N Engl J Med.

[21] Siegfried N, Muller M, Deeks JJ, Volmink J (2009). Male circumcision for prevention of heterosexual acquisition of HIV in men. Cochrane Database Syst Rev.

[22] Bailey RC, Moses S, Parker CB (2007). Male circumcision for HIV prevention in young men in Kisumu, Kenya: a randomised controlled trial. Lancet.

[23] Gray RH, Kigozi G, Serwadda D (2007). Male circumcision for HIV prevention in men in Rakai, Uganda: a randomised trial. Lancet.

[24] Thirumurthy H, Masters SH, Rao S (2014). Effect of providing conditional economic compensation on uptake of voluntary medical male circumcision in Kenya: a randomized clinical trial. JAMA.

[25] Solomon SS, Srikrishnan AK, Vasudevan CK (2014). Voucher incentives improve linkage to and retention in care among HIV-infected drug users in Chennai, India. Clin Infect Dis.

[26] Kennedy CE, Yeh PT, Atkins K (2020). Economic compensation interventions to increase uptake of voluntary medical male circumcision for HIV prevention: a systematic review and meta-analysis. PLoS One.

[27] Dovel K, Shaba F, Offorjebe OA (2020). Effect of facility-based HIV self-testing on uptake of testing among outpatients in Malawi: a cluster-randomised trial. Lancet Glob Health.

[28] Choko AT, MacPherson P, Webb EL (2015). Uptake, accuracy, safety, and linkage into care over two years of promoting annual self-testing for HIV in Blantyre, Malawi: a community-based prospective study. PLoS Med.

[29] Simwinga M, Kumwenda MK, Dacombe RJ (2019). Ability to understand and correctly follow HIV self-test kit instructions for use: applying the cognitive interview technique in Malawi and Zambia. J Int AIDS Soc.

[30] Carballo-Diéguez A, Giguere R, Balán IC (2020). Use of rapid HIV self-test to screen potential sexual partners: results of the ISUM study. AIDS Behav.

[31] MacGowan RJ, Chavez PR, Borkowf CB (2020). Effect of internet-distributed HIV self-tests on HIV diagnosis and behavioral outcomes in men who have sex with men: a randomized clinical trial. JAMA Intern Med.

[32] Wu D, Zhou Y, Yang N (2020). Social media-based secondary distribution of HIV/syphilis self-testing among Chinese men who have sex with men. Clin Infect Dis.

