# Supplementary material for: Partner-delivered HIV self-test kits with and without financial incentives in antenatal care and index patients with HIV in Malawi: a three-arm, cluster-randomised controlled trial
Source: Lancet Glob Health. 2021 Jun 15;9(7):e977–88. doi: 10.1016/S2214-109X(21)00175-3 (PMC8220130; doi:10.1016/S2214-109X(21)00175-3)
Supplement: Supplementary appendix [file mmc1.pdf]

THE LANCET
Global Health

Supplementary appendix

This appendix formed part of the original submission and has been peer reviewed. We post it as supplied by the authors.

Supplement to: Choko AT, Fielding K, Johnson CC, et al. Partner-delivered HIV self-test kits with and without financial incentives in antenatal care and index patients with HIV in Malawi: a three-arm, cluster-randomised controlled trial. *Lancet Glob Health* 2021; **9:** e977–88.

APPENDIX: Figure 1: Summary of male partner testing, cluster-by-cluster (intention-to-treat analysis)

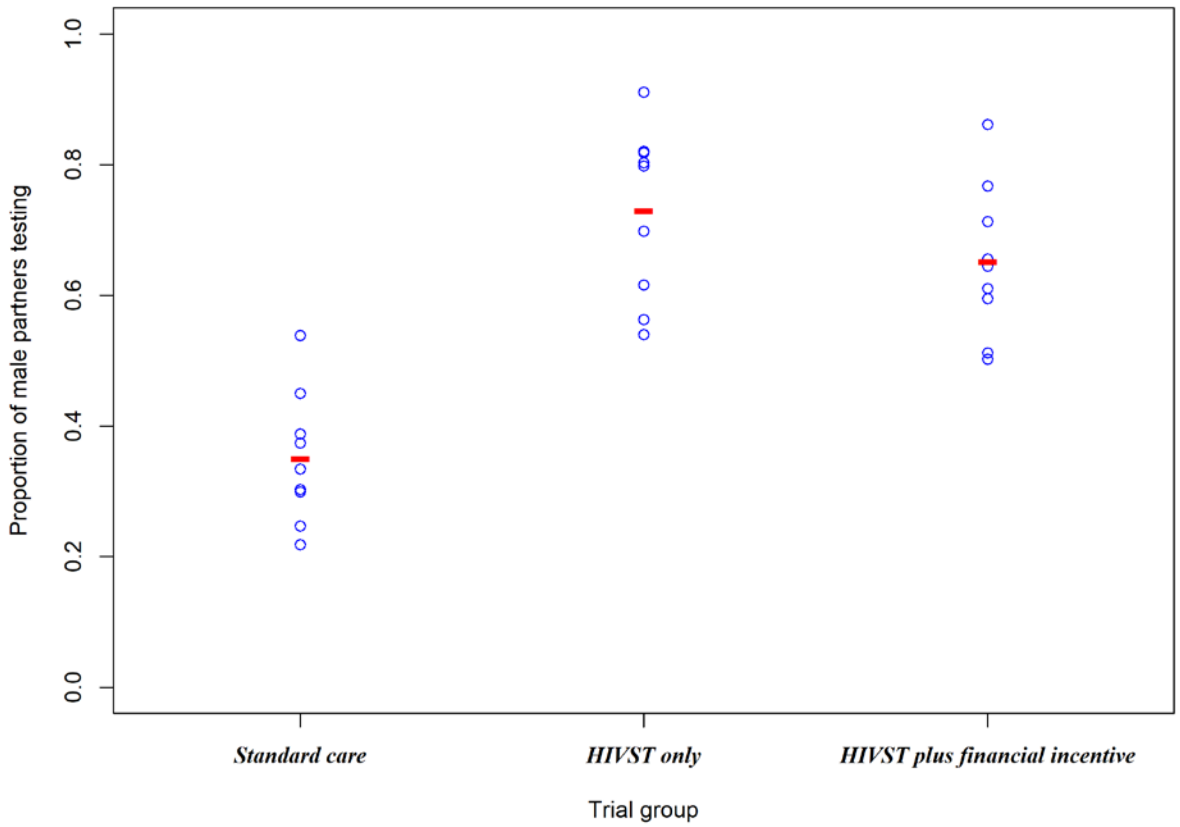

HIVST: HIV self-testing. Each blue circle represents the proportion of male partners testing at a clinic. Red line represents the geometric mean for each arm.

APPENDIX: Figure 2: Summary of new HIV positive sexual contacts identified

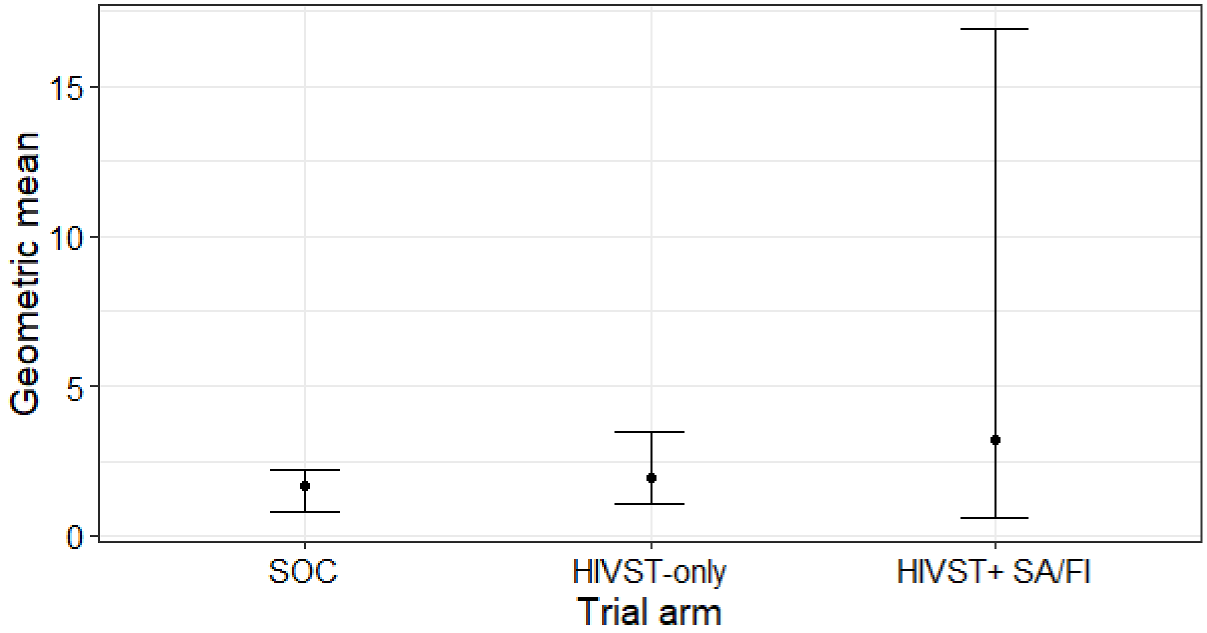

Bars represent confidence interval for the geometric mean

SOC: standard of care; HIVST: HIV self-testing; SA/FI: secondary accuracy/financial

APPENDIX: Figure 3: Percentage of all partners linking to HIV treatment or circumcision* (*n* =4,544 eligible women).

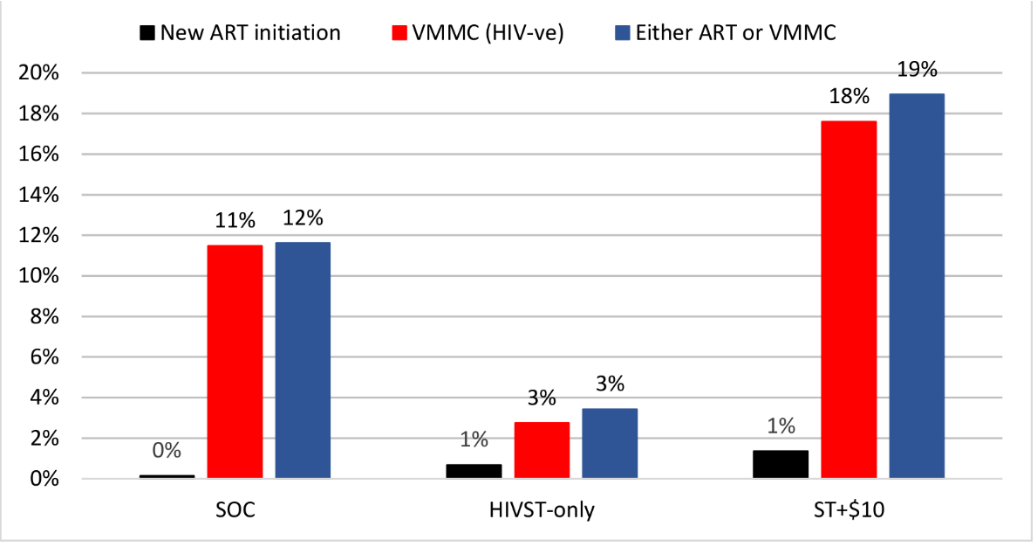

Intention-to-treat analysis using the denominator of all eligible women and index patients.
* Includes VMMC booking, even if procedure not carried out.
ART, antiretroviral therapy; VMMC, voluntary medical male circumcision; SOC: standard of care; HIVST: HIV self-testing; SA/FI: secondary accuracy/financial
In the HIVST-only arm, only those with a positive self-test were encouraged to attend the clinic.
